# Case Report: Repeated Stereotactic Radiotherapy of Recurrent Ventricular Tachycardia: Reasons, Feasibility, and Safety

**DOI:** 10.3389/fcvm.2022.845382

**Published:** 2022-03-28

**Authors:** Jana Haskova, Petr Peichl, Marek Šramko, Jakub Cvek, Lukáš Knybel, Otakar Jiravský, Radek Neuwirth, Josef Kautzner

**Affiliations:** ^1^Department of Cardiology, Institute for Clinical and Experimental Medicine (IKEM), Prague, Czechia; ^2^Department of Oncology, University Hospital Ostrava, Ostrava, Czechia; ^3^Department of Oncology, Ostrava University Medical School, Ostrava, Czechia; ^4^Department of Cardiology, Podlesí Hospital Trinec, Trinec, Czechia; ^5^Department of Cardiology, Masaryk University Medical School, Brno, Czechia

**Keywords:** stereotactic body radiotherapy, ventricular tachycardia, electroanatomical mapping, failed catheter ablation, safety

## Abstract

Stereotactic body radiotherapy (SBRT) has been reported as an attractive option for cases of failed catheter ablation of ventricular tachycardia (VT) in structural heart disease. However, even this strategy can fail for various reasons. For the first time, this case series describes three re-do cases of SBRT which were indicated for three different reasons. The purpose in the first case was the inaccuracy of the determination of the treatment volume by indirect comparison of the electroanatomical map and CT scan. A newly developed strategy of co-registration of both images allowed precise targeting of the substrate. In this case, the second treatment volume overlapped by 60% with the first one. The second reason for the re-do of SBRT was an unusual character of the substrate–large cardiac fibroma associated with different morphologies of VT from two locations around the tumor. The planned treatment volumes did not overlap. The third reason for repeated SBRT was the large intramural substrate in the setting of advanced heart failure. The first treatment volume targeted arrhythmias originating in the basal inferoseptal region, while the second SBRT was focused on adjacent basal septum without significant overlapping. Our observations suggested that SBRT for VT could be safely repeated in case of later arrhythmia recurrences (i.e., after at least 6 weeks). No acute toxicity was observed and in two cases, no side effects were observed during 32 and 22 months, respectively. To avoid re-do SBRT due to inaccurate targeting, the precise and reproducible strategy of substrate identification and co-registration with CT image should be used.

## Introduction

Current strategies of catheter ablation are effective in the prevention of recurrences of ventricular tachycardias (VTs) (1–3). Not frequently, catheter ablation may fail due to the inability to reach the critical part of the substrate (4, 5). The reasons include deep intramural location or failure to negotiate epicardial access (usually after previous surgery). Among the alternative treatment strategies, stereotactic body radiotherapy (SBRT) was first reported in case reports or case series as an attractive option (6–8).

The experience with SBRT is gradually growing and several other case reports and prospective clinical studies documented a significant decrease in VT occurrences (9–19). However, even this strategy can fail for various reasons. Hence, the goal of this report was to describe a case series of re-do SBRT for VT recurrences, which is the first time in the literature.

## Methods

Since we used the same strategy of SBRT in all sessions, a brief description was provided here. The MultiPlan treatment planning system with sequential dose optimization and the CyberKnife radiosurgery system (both from Accuray, Inc., Sunnyvale, CA, USA) were employed as described previously (12). After image registration with two ECG-gated CT scans (in both systole and diastole), the internal target volume was calculated to account for heart contractions. For compensation of respiratory movements, the existing implantable cardioverter-defibrillator (ICD) lead was used as a surrogate marker. The tracking mode relevant to SBRT for VT is Synchrony using “fiducials”. Based on the target surrogate, which is an ICD lead tip, in this case, a correlation respiratory motion model was created before the treatment. Such model was based on lead 3D locations extracted from a series of X-ray image pairs and corresponding respiratory phase signals from LED markers placed on the patient's chest. The created model was then used during dose delivery to control radiation source position and orientation to move together with the target (surrogate) while the beam was on. During treatment, the correlation model was updated with every new pair of X-ray images. In principle, this technology required minimum target volume expansion to cover respiratory motion-related target position variation during breathing so it has relatively better potency to spare normal tissue from dose.

## Case Series

### Case 1

**Figure 1:**
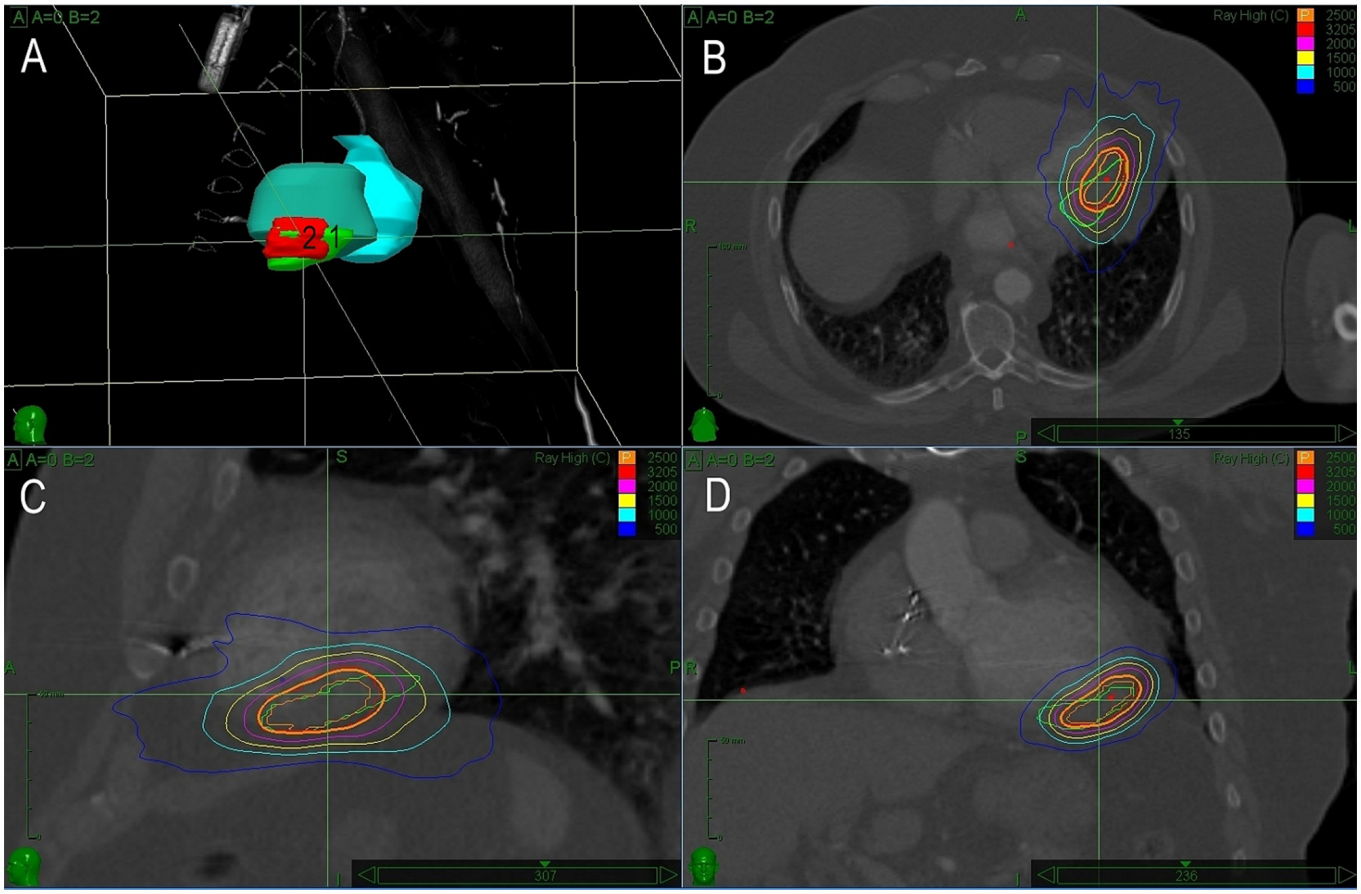
A treatment plan for Case 1. **(A)** 3D reconstruction of planned treatment volumes for the first (1) and second (2) radiotherapy. **(B–D)** Depict sagittal, coronal, and axial views with isodose lines for both sessions of radiotherapy (green line shows target volume for the first and red for the second session). In this case, there is a significant overlap of both treatment volumes caused by inaccuracy in the planning of the first session.

The first case was reported recently in detail as a case report, illustrating the need for precision in planned target volume (PTV) determination (20). Briefly, a 66-year-old man with a history of coronary artery bypass graft surgery and primary prophylactic ICD implant (left ventricular ejection fraction of 35%) underwent catheter ablation for recurrences of slow VT. Clinical VT originated from a small reentrant circuit located intramurally and/or epicardially below the base of the posteromedial papillary muscle. Despite multiple endocardial ablation attempts, VT remained inducible and an attempt for percutaneous epicardial approach failed because of severe adhesions from previous cardiac surgery. The first SBRT session was planned based on a visual alignment of the presumed origin of VT from electroanatomical maps and CT images. A single fraction of 25Gy was delivered. For recurrences of VT episodes of the same ECG morphology, the patient underwent the second electrophysiology study and remapping 14 months later. Based on the electroanatomical mapping, the low voltage area caused by the previous SBRT was adjacent to the site of the earliest endocardial activity during VT. Additional RF ablation failed again to prevent the inducibility of VT and we used a newly developed co-registration method for the precise targeting of the SBR (20). Detailed maps were presented in a previously published case report (20). Briefly, there was only a small bipolar low voltage area after the first SBRT which was adjacent to the true exit of VT. Precise co-registration of the target in the second SBRT allowed to establish a smaller PTV amounting to 18 ml, including an additional 3 mm margin. The dice overlap of previous and new PTV was 0.68. The second session was performed 19 months after the first one. The same dose of 25 Gy was delivered (Figure 1). After transient early recurrences of slow VT, arrhythmias gradually disappeared within 3 months and the patient became arrhythmia-free for 32 months. No adverse effect of SBRT was observed during this period.

### Case 2

**Figure 2:**
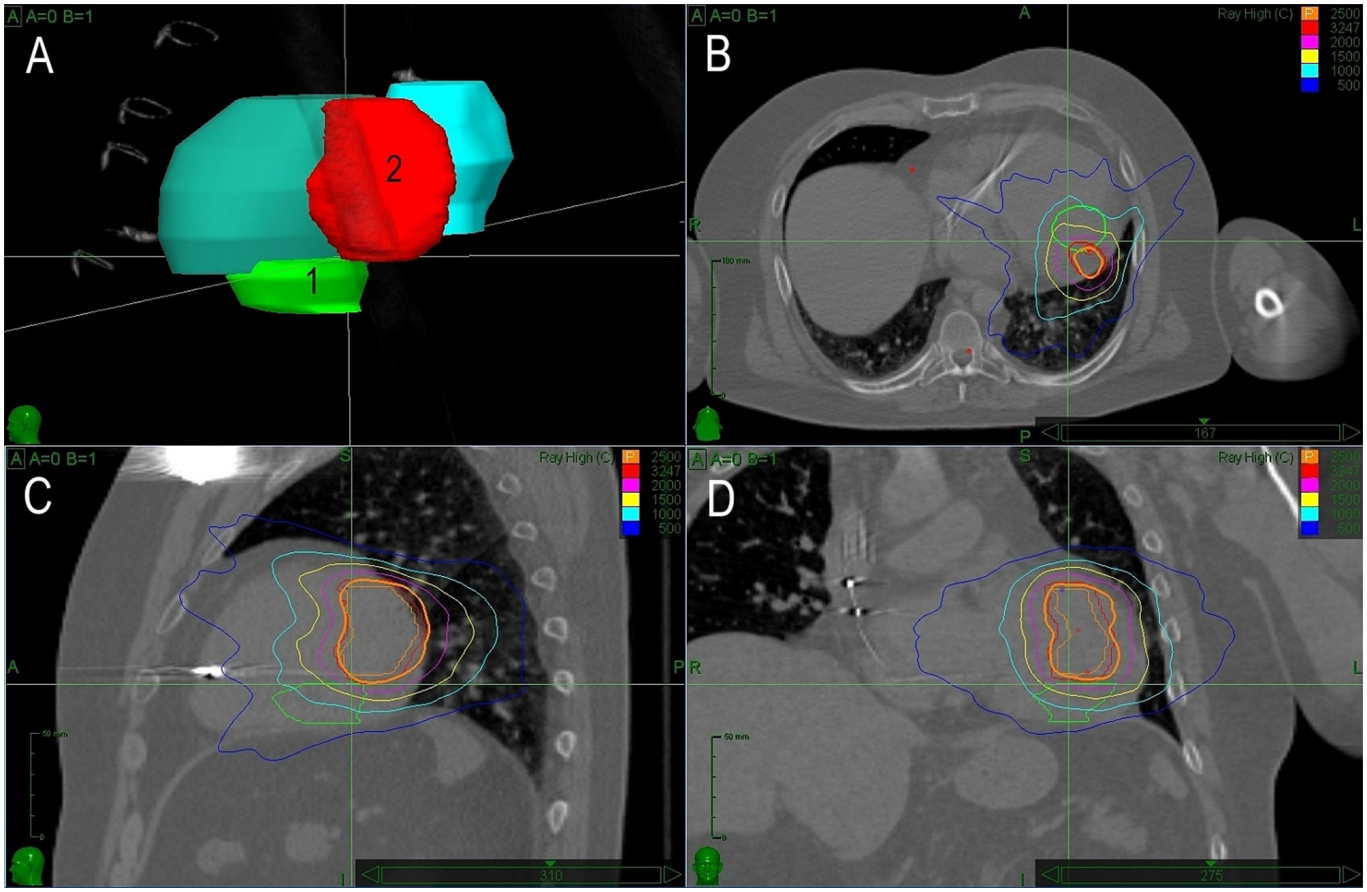
The treatment plan for Case 2. Description of **(A–D)** is identical as in Figure 1. In this case, the second planned target volume covers an entirely different region.

The second case of a patient with cardiac fibroma triggering recurrent VTs of different morphologies was reported after the first successful SBRT in 2017 (10). Briefly, it was a 34-year-old patient diagnosed with an intramyocardial tumor (60 × 40 × 25 mm) located in the inferolateral wall of the left ventricle. The patient presented with several morphologies of VT. The patient underwent exploratory surgery, but the excision of the tumor was impossible for its size. Only far-field signals were recorded above and around the tumor. Empirical epicardial cryoablation around the tumor was performed with transient suppression of VTs. Subsequent electroanatomical mapping and pace mapping identified two regions responsible for two residual clinical VTs. One had a reentrant character with an exit in the lateral wall, which was close to the summit. This VT was non-inducible after catheter ablation. The other VT became incessant and originated in a region between the septum and posteromedial papillary muscle. It had characteristics of focal VT with a source located deep in the wall, adjacent to the tumor. The patient was referred for SBRT. PTV was determined based on tumor location and visual comparison with electroanatomical maps. SBRT was performed with 25 Gy to the 75% isodose line. After the procedure, VT disappeared gradually within 6 months. The patient was without any arrhythmia for the next 22 months. However, the patient remained on amiodarone which had to be stopped due to amiodarone-related thyrotoxicosis. After successful treatment of this condition, the patient was without arrhythmias for the next 10 months. Then, the patient returned with an electrical storm and one morphology of VT. Electrophysiological study induced sustained VT from the anterolateral basal part of the ventricle. Electroanatomical bipolar voltage map showed normal values and pacing revealed slowed conduction in this region. Ablation did not prevent the inducibility of VT due to the deep location of the substrate. The second session of SBRT was planned and conducted based on precise integration of data from electroanatomical mapping and CT. PTV for the second SBRT was applied on the opposite side of the tumor and there was no overlap with the first radiotherapy site. The size of the tumor remained the same. After the second SBRT (25 Gy, PTV 62.2 ml; Figure 2), the patient remained without VTs and did not gain any adverse effects for 22 months.

### Case 3

**Figure 3:**
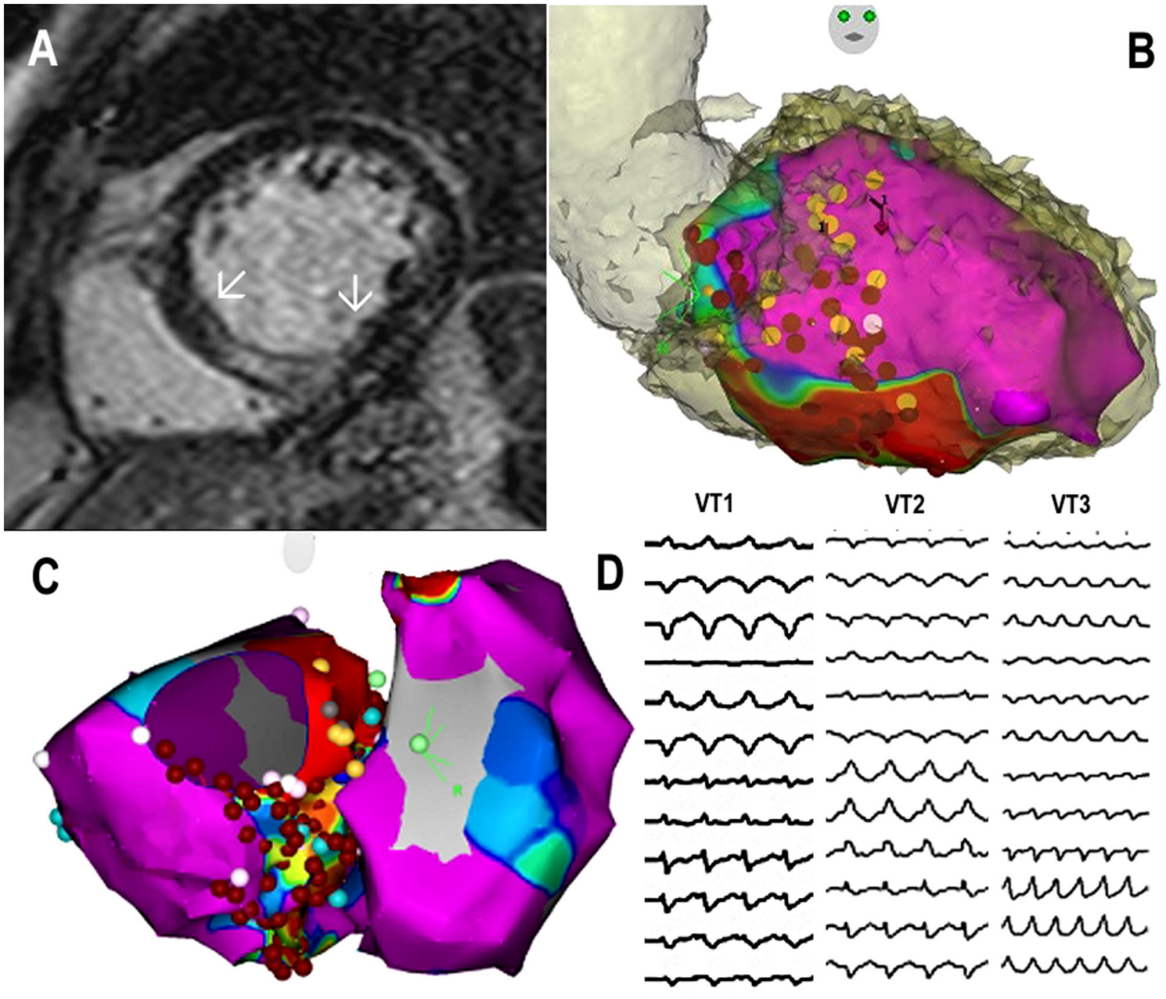
Clinical findings from Case 3: **(A)** MRI with late gadolinium enhancement within the basal septum and adjacent inferior wall (arrows); **(B)** electroanatomical bipolar voltage map merged with CT angiogram of the left ventricle in right anterior oblique (RAO) view, displaying low voltage inferoseptally and ablation points in this region and at midseptal level before 1st stereotactic body radiotherapy (SBRT); **(C)** electroanatomical bipolar voltage maps obtained during third catheter ablation before 2nd SBRT, showing a larger area of low voltage in the entire basal septum up to outflow tract with ablation points; **(D)** ECG recordings of dominant ventricular tachycardia (VT) morphologies: VT1 and VT2 before the first SBRT, VT3 before the second SBRT. Note change in the exit from the low septum to high septum for VT3.

**Figure 4:**
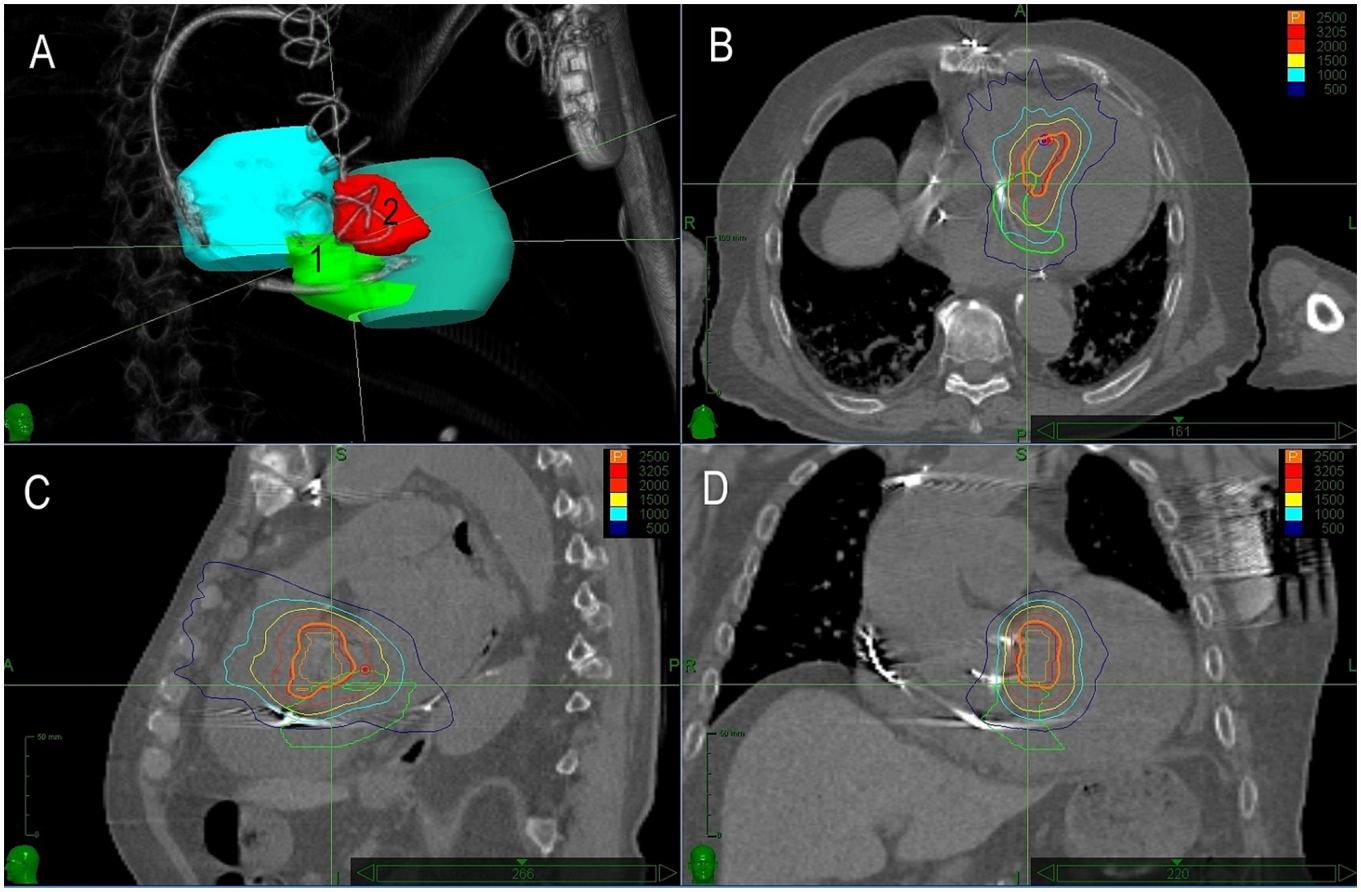
The treatment plan for Case 3. Description of **(A–D)** is identical as in Figure 1. In this case, the second planned volume covered extensive substrate within the basal septum.

The third patient was a 77-year-old man with a diagnosis of non-ischemic cardiomyopathy and intramural location of fibrosis in the basal region of the left ventricle. The patient presented with ventricular arrhythmias for several years and was implanted with a single chamber ICD. Later, aortic valve replacement with mechanical prosthesis was performed for aortic regurgitation together with a concomitant MAZE procedure. After 3 years, the device was upgraded to cardiac resynchronization therapy-defibrillators (CRT-D). At that time, the patient presented with an electrical storm. They underwent electroanatomical mapping and substrate ablation in the inferoseptal region of the left ventricle two times. For sporadic recurrences of VT, the patient was referred for SBRT 1 month later (Figure 3). A 25 Gy dose was applied to the basal inferoseptal region. After temporary improvement, the patient presented with recurrent VTs and underwent 2 months later another electrophysiology study. Three different VTs were induced, all with the exit in the septum above the initially irradiated region. The entire basal septum showed decreased bipolar voltage and catheter ablation covered it all. No VT was inducible at the end of the procedure. The patient was readmitted for decompensated heart failure due to incessant VT with an exit in the upper septum and was indicated to re-do SBRT. The septal region adjacent to the initial PTV was delineated as a new PTV with minimal spatial overlap. The second SBRT was performed 4 months after the first one (Figure 4). After SBRT, the patient continued to present with slow VT (CL around 600 ms) which necessitated another catheter ablation from both sides of the interventricular septum. Non-inducibility of VT was achieved. Although the patient was without VT, their overall clinical status gradually deteriorated and then eventually died due to the progression of heart failure 1 month later. No autopsy was performed.

Timelines of treatment for all three patients are listed in Table 1. Dose-volume parameters of organs at risk (OAR) and PTVs are enumerated in Table 2.

**Table 1:** Timelines.

**Case 1**	
Index date	A 66-year-old male with ischemic cardiomyopathy and recurrent VTs requiring therapy from ICD
1,5,11 months	Repeated ineffective catheter ablations due to intramural location of the substrate
18 months	First SBRT with continuing recurrences of VT
34 months	Remapping after the first SBRT
38 months	Second SBRT, after 3 months VT disappeared
69 months	Last follow-up visit, no arrhythmias
**Case 2**	
Index date	A 34-year-old patient with an intramyocardial fibroma (60 × 40 × 25 mm) in the inferolateral wall of the left ventricle and recurrent VTs of different morphologies
6 months	Empirical circumferential epicardial cryoablation around the tumor, 6 months without recurrences of VT
13 months	Catheter ablation for recurrences of 2 morphologies of VT, one non-inducible, other almost incessant
14 months	First SBRT, within 6 months all arrhythmias gradually disappeared
38 months	v Re-do catheter ablation, without elimination of VT due to intramural substrate located in the opposite side of the tumor
38 months	Second SBRT, within 3 months VT disappeared
60 months	Last follow-up visit, no arrhythmias
**Case 3**	
Index date	A77-year-old male with non-ischemic cardiomyopathy, aortic valve replacement and fibrosis in basal region of left ventricle and sporadic interventions of ICD
70 months	Repeated catheter ablation for electrical storm
71 months	First SBRT, arrhythmias less frequent
73 months	Re-do catheter ablation for VT recurrences in basal septal region above the previous SBRT, non-inducibility
75 months	Second SBRT for incessant VT, leading to slowing VT to 100 bpm
76 months	Re-do catheter ablation in the basal septum, non-inducibility
77 months	Progression of heart failure and cachexia, death

**Table 2:** Parameters of organs at risk (OAR) and planning target volume (PTV).

**OAR and PTV volume parameter**	**Case 1**	**Case 2**	**Case 3**
Heart D15 ml (Gy)	46.3	42.4	42.9
Heart D0,035 ml (Gy)	61.0	51.3	50.0
Heart Dmean (Gy)	4.8	13.5	14.5
Lung Left Dmean (Gy)	1.8	7.7	2.1
Esophagus D5 ml (Gy)	5.9	4.9	13.3
Esophagus D0,035 ml (Gy)	8.6	7.1	21.9
Stomach D10 ml (Gy)	10.4	7.3	6.5
Stomach D5 ml (Gy)	12.2	8.0	8.7
Stomach D0,035 ml (Gy)	18.3	11.6	14.3
PTV (mL)	21.2	23.4	43.4
PTVredo (mL)	18.3	62.2	20.0
PTVxPTVredo (mL)	11.3	0.1	0.4
PTVxPTVredo (%)	61.7	0.2	2.2
PTVxPTVredo Dmax (Gy)	32.1	25.6	25.8
PTVxPTVredo D0,035 ml(Gy)	31.9	24.5	25.6
PTVxPTVredo Dmean (Gy)	28.8	24	25.2

*Dose-volume parameters are based on integrated isodose plans calculation from the first and second SBRT sessions. Both CT series from simulation were registered according to the heart region and summation of dose distribution was performed. D—abbreviation for dose; D5 ml, D10 ml, and D15 ml represents the dose to 5, 10, and 15 ml of relevant OAR, respectively. D.035 ml represents near-maximum dose, “x” means the intersection of volumes, redo means second irradiation*.

## Discussion

This case series is the first of this kind that reports on the feasibility and acute and mid-term safety of re-do SBRT in patients with recurrent VTs. The reason for repeated SBRT was different in all three subjects. One reason was the inaccuracy of targeting when using indirect comparison of electroanatomical maps with pretreatment CT. The second reason for re-do SBRT was an unusual character of the substrate, wherein there is an inoperable cardiac fibroma associated with several morphologies of VT from different regions of the tumor. The third reason for repeated SBRT was the large intramural basal septal substrate in the setting of dilated cardiomyopathy and advanced heart failure.

Regarding the safety of re-do SBRT, it is important to keep in mind that the risk of cardiovascular complications associated with chest radiotherapy can persist for many years (21, 22). Studies on the relationship between the dose and adverse outcomes show up to 16% relative risk of heart disease and major cardiac events per Gy of the mean heart dose (23, 24). In addition, other studies showed correlations between radiation doses to specific cardiac regions and cardiac morbidity and mortality (21). In the case of repetition of SBRT, the likelihood of severe toxicity may increase. Since no radiation-related adverse events were observed in our patients, we were not able to comment about the relationship between the dose to organs at risk dose and the occurrence of side effects.

Another fear may concern the further worsening of left ventricular ejection fraction after the second SBRT. Importantly, we did not observe a significant change in this parameter nor the significant increase of cardiac troponin after the second SBRT. Additionally, our patients had no clinical symptoms or signs of pericarditis or pneumonitis. The third patient died of terminal heart failure which was not in our opinion in relation to the second SBRT. One explanation for the good tolerability of the repeated SBRT may reflect the fact that our strategy of SBRT uses relatively small PTV, covering the critical region of the substrate (12).

We found only one case of re-do SBRT description in the literature. It was in a series by Lloyd et al. (15) who reported on outcomes of SBRT in 10 patients with advanced heart failure and VTs. One patient in this group who had no response to SBRT underwent a second SBRT ineffective treatment 90 days later. The patient was considered an outlier and ultimately underwent heart transplantation for recurrent VTs despite all therapies. No more details were available.

For a discussion on the indication to re-do SBRT and its safety, it is important to recall that the tissue effect of SBRT for VT in humans remains largely unknown, and also the time window to clinical effect is highly variable. Most of the experimental studies suggested that electrophysiological effects are rather delayed and that the development of fibrosis is important for clinical effect (25, 26). Our recent report analyzing 3 post-mortem hearts after SBRT is in line with the above experimental data on early apoptosis and delayed fibrosis (27). Furthermore, the clinical effect of SBRT was delayed and a similar pattern was observed also in the current series (28, 29). In the largest published clinical study of Encore VT, the blanking period of 6 weeks was used to avoid counting early recurrences of VT (9). It appeared that in the majority of cases, the clinical effect should be observed after 2–3 months. Later recurrences or incessant VT could be considered either for re-do catheter ablation or repeated SBRT.

However, anecdotal cases described the immediate clinical effect of SBRT resulting in acute termination of an electrical storm (11, 13, 14, 16). Some recent experimental studies suggested that the clinical effect of SBRT is not necessarily related to the development of fibrosis and even deconstruct fibrosis as the main antiarrhythmic mechanism. A study by Zhang DM et al. demonstrated that postmortem heart specimens from four patients, with a substantial reduction of VT after SBRT, did not exhibit transmural fibrosis within the timeframe of VT reduction (30). In an experimental study, electrophysiologic assessment of irradiated murine hearts revealed a persistent supraphysiologic electrical phenotype, mediated by increases in components of the Natrium channel and Cx43. Additionally, increased Na_V_1.5 expression was also found in the explanted human heart from the said clinical study. The authors offered an alternative explanation of the effect of SBRT—increased cardiac conduction. Interestingly, another experimental study suggested a different mechanism for the early effect of SBRT (31). In the rat model of SBRT, the authors found acute structural changes, such as interstitial and subsarcolemmal edema, widening of intercalated discs, and microvascular inflammatory responses. These acute structural changes resulted in the slowing of intracardiac conduction on ECG, which might be an alternative explanation of the effect of SBRT. These observations may suggest that an even shorter time window than 2–3 months could be employed to consider a failure of SBRT and re-do procedure.

Our first case emphasized the need for using the accurate and reproducible strategy of planned target volume delineation. Using the novel method of co-registration of electroanatomical maps with pretreatment CT, we were able to correct the previous treatment plan and deliver successfully therapy (20). More recently, we showed reproducibility of this strategy (32). The other important issue related to accuracy and safety is how to minimalize the treatment volume with respiratory compensation. We used ICD lead tracking as described above. The other possibility is the use of continuous real-time imaging and tracking of the moving target during treatment with gated irradiation using MR-guided radiotherapy (33). Therefore, with current strategies of accurate targeting of the critical substrate region and motion mitigation, the main reason for considering re-do SBRT should be either extensive substrate or development of a new substrate in a different region of the heart.

## Conclusion

Our observations suggest that SBRT for VT could be repeated in case of arrhythmia recurrences with good acute and mid-term safety. Long-term safety remains to be further documented. The clinical effect of SBRT appears to be predominantly delayed and re-do procedures should be considered after a 2–3 month period. For earlier indications, there is still limited evidence. With current strategies of accurate targeting of the critical substrate region and precise delivery of SBRT, the main reason for considering re-do SBRT should be either extensive substrate or development of the new substrate in a different region of the heart.

## Data Availability Statement

The original contributions presented in the study are included in the article/supplementary material, further inquiries can be directed to the corresponding author.

## Ethics Statement

The studies involving human participants were reviewed and approved by Ethical Committee of the Institute for Clinical and Experimental Medicine, Prague, Czechia. The patients/participants provided their written informed consent to participate in this study. Written informed consent was obtained from the individual(s) for the publication of any potentially identifiable images or data included in this article.

## Author Contributions

JH: preparation of the manuscript and organization. PP: catheter ablation, organization, and correction of manuscript. MŠ: development of co-registration strategy and correction of manuscript. JC: radiotherapist and reading manuscript. LK: radiotherapy planning and figure preparation. OJ: catheter ablation. RN: organization, leading part of project, and preparation of manuscript. JK: catheter ablation, leading project, and final corrections to manuscript. All authors contributed to the article and approved the submitted version.

## Funding

This work was supported by grant project AZV NU20-02-00244 from the Ministry of Health of the Czech Republic and by funding from the European Union's Horizon 2020 research and innovation program under grant agreement No 945119.

## Conflict of Interest

JH received speaker honoraria from ProMed, PP has received speaker honoraria from Abbott and ProMed. JK reports personal fees from Bayer, Biosense Webster, Boehringer Ingelheim, Medtronic, and Abbott for participation in scientific advisory boards, and has received speaker honoraria from Bayer, Biosense Webster, Biotronik, Boehringer Ingelheim, Medtronic, Mylan, Pfizer, ProMed, and Abbott. The remaining authors declare that the research was conducted in the absence of any commercial or financial relationships that could be construed as a potential conflict of interest.

## Publisher's Note

All claims expressed in this article are solely those of the authors and do not necessarily represent those of their affiliated organizations, or those of the publisher, the editors and the reviewers. Any product that may be evaluated in this article, or claim that may be made by its manufacturer, is not guaranteed or endorsed by the publisher.
